# Association between a multi-component web-based mental health intervention and clinical outcomes in patients with psychological disorders: a historical controlled study

**DOI:** 10.3389/fpsyg.2026.1781089

**Published:** 2026-03-09

**Authors:** Jingfeng Cheng, Yuhong Wang, Cuiqing Tang

**Affiliations:** 1 Department of Administrative Management, The Third People’s Hospital of Qingyuan, Qingyuan, China; 2 Department of Psychiatry, The Third People’s Hospital of Qingyuan, Qingyuan, China

**Keywords:** anxiety, assessment, depression, mental health, quality of life, sleep, web-based system

## Abstract

**Objective:**

To evaluate the association between a multi-component web-based mental health intervention and clinical outcomes in patients with psychological disorders through a historical controlled study.

**Methods:**

This historical controlled study included 483 patients diagnosed with psychological disorders who received interventions at our hospital between August 2022 and October 2024. Patients were divided into a control group (CG, *n* = 238, treated from August 2022 to June 2023 using conventional paper-based assessments) and a study group (SG, *n* = 245, treated from July 2023 to October 2024 with multi-component web-based mental health intervention). Both groups received standardized treatment according to clinical practice guidelines, with the primary difference being the assessment modality. The web-based system enabled remote completion of standardized assessments and provided real-time data visualization for clinicians. Assessment indicators included the Patient Health Questionnaire-9 (PHQ-9), Generalized Anxiety Disorder-7 (GAD-7), Pittsburgh Sleep Quality Index (PSQI), 36-Item Short Form Health Survey (SF-36), Social Disability Screening Schedule (SDSS), and World Health Organization Quality of Life-BREF (WHOQOL-BREF). Follow-up assessments were conducted at baseline and at 1, 3, and 6 months post-intervention.

**Results:**

Baseline characteristics including gender distribution, mean age, mean BMI, and types of psychological disorders were comparable between groups (*p* > 0.05). At 1-month, 3-month, and 6-month follow-ups, the study group demonstrated significantly lower PHQ-9, GAD-7, and PSQI scores compared to the control group (*p* < 0.05). At 6 months post-intervention, the study group exhibited higher scores across all SF-36 dimensions (physical functioning, role-physical, bodily pain, general health, vitality, social functioning, role-emotional, and mental health) compared to the control group (*p* < 0.05), with a total score of 74.53 ± 9.94, significantly higher than the control group’s 63.70 ± 9.89 (*t* = 11.997, *p* < 0.001). Additionally, at 6 months post-intervention, the study group had significantly lower SDSS scores and higher WHOQOL-BREF scores than the control group (*p* < 0.05).

**Conclusion:**

This historical controlled study observed an association between a multi-component web-based mental health intervention and improvements in depression, anxiety, sleep symptoms, quality of life, and social functioning. Given the historical design and pandemic-related temporal confounding, these findings should be interpreted as exploratory and hypothesis-generating.

## 1 Introduction

According to the World Health Organization, approximately 970 million people worldwide suffer from mental disorders, with depression and anxiety being the most common mental health problems, affecting around 280 million and 301 million people, respectively (Ang et al., 2025). In China, the Seventh National Population Census data indicates that the lifetime prevalence of psychological disorders has reached 16.6%, with depressive disorders, anxiety disorders, and sleep disorders ranking highest (Arrow et al., 2023). Mental health problems not only severely affect patients’ quality of life and social functioning but also impose a heavy burden on families and society.

Currently, mental health assessment methods commonly used in hospitals primarily rely on traditional paper questionnaires and face-to-face clinical interviews. While these methods have certain validity, they have obvious limitations: paper questionnaires are cumbersome to manage, time-consuming to process, and difficult to analyze in real-time; face-to-face assessments are constrained by time and location, making it difficult to meet patients’ needs for continuous monitoring; traditional assessment methods struggle to overcome information concealment due to stigmatization concerns; furthermore, uneven distribution of medical resources makes quality mental health services difficult to access in remote areas. These issues seriously restrict the accessibility and effectiveness of mental health services (Chamorro-Delmo et al., 2024; Cui et al., 2022).

With the rapid development of internet technology, integrating information technology into mental health assessment systems demonstrates significant advantages. Web-based assessment systems enable real-time data collection and analysis, providing timely support for clinical decision-making; patients can conduct self-assessments at any time and place, greatly enhancing the convenience of assessment; electronic systems can automatically remind follow-ups, ensuring treatment compliance; digital data management facilitates long-term tracking of symptom changes, enabling precise interventions; additionally, the anonymity of online platforms helps reduce patients’ psychological burden of self-disclosure, improving information authenticity (Curelaru and Curelaru, 2024; Fischer et al., 2025).

To address the limitations of traditional mental health assessment and harness the potential of digital technology, we developed a multi-component web-based mental health intervention and implemented it in clinical practice. The system enables patients to complete standardized assessments remotely while providing clinicians with real-time access to multidimensional data on symptom severity, quality of life, and social functioning. Furthermore, this system could automatically remind patients to complete scheduled follow-up. In this controlled clinical study involving 483 patients across six common psychological disorders, we examined whether this digital approach could be associated with differences in treatment-related outcomes compared with conventional care. The findings offer preliminary insights into the potential role of technology-enabled assessment in modern mental health service delivery and its potential to improve patient outcomes in an increasingly digital healthcare landscape.

## 2 Research design and methods

### 2.1 Research design and process

This research was a historical controlled study, approved by the hospital ethics committee 2023EA089, and complied with the requirements of the Declaration of Helsinki. The study period was set from August 2022 to October 2024, with samples sourced from Qingyuan Third People’s Hospital. The control group consisted of patients who received conventional treatment from August 2022 to June 2023, while the study group consisted of patients who received treatment assisted by multi-component web-based mental health intervention from July 2023 to October 2024. Initial patient screening was conducted through the hospital’s HIT system according to the timeframe and the following inclusion criteria:

Inclusion criteria: (1) Patients diagnosed with anxiety disorder, depressive disorder, sleep disorder, adjustment disorder, post-traumatic stress disorder, or obsessive-compulsive disorder according to the Chinese Classification and Diagnostic Criteria of Mental Disorders, Third Edition (CCMD-3) or International Classification of Diseases, Tenth Revision (ICD-10) (Uysal, 2019); (2) Age 18–65 years; (3) Received systematic intervention at the hospital.

After screening according to the above inclusion criteria, a total of 536 patients entered the study, followed by secondary screening according to exclusion criteria:

Exclusion criteria: (1) Concurrent serious physical diseases (such as malignant tumors, severe cardiovascular diseases, uncontrolled diabetes, etc.); (2) Previous diagnosis of schizophrenia, bipolar disorder, organic mental disorder, or intellectual disability; (3) Patients who received electroconvulsive therapy (ECT) or transcranial magnetic stimulation (TMS) during the study period; (4) Incomplete scale assessment data (more than 1 scale missing) at any follow-up time points; (5) Pregnant women, lactating women, and patients with a history of alcohol or drug abuse; (6) Study group patients who could not normally use the multi-component web-based mental health intervention due to technical barriers (such as inability to operate smart devices or connect to the internet).

After screening according to the exclusion criteria, a total of 483 patients were included in the study, with 238 in the control group and 245 in the study group. Both groups received standardized treatment according to clinical practice guidelines for their respective diagnoses, including appropriate pharmacological interventions and psychological therapies. The key difference between groups was the assessment method: the study group utilized the multi-component web-based mental health intervention for symptom monitoring and data collection, while the control group used traditional paper-based questionnaires.

**Figure 1:**
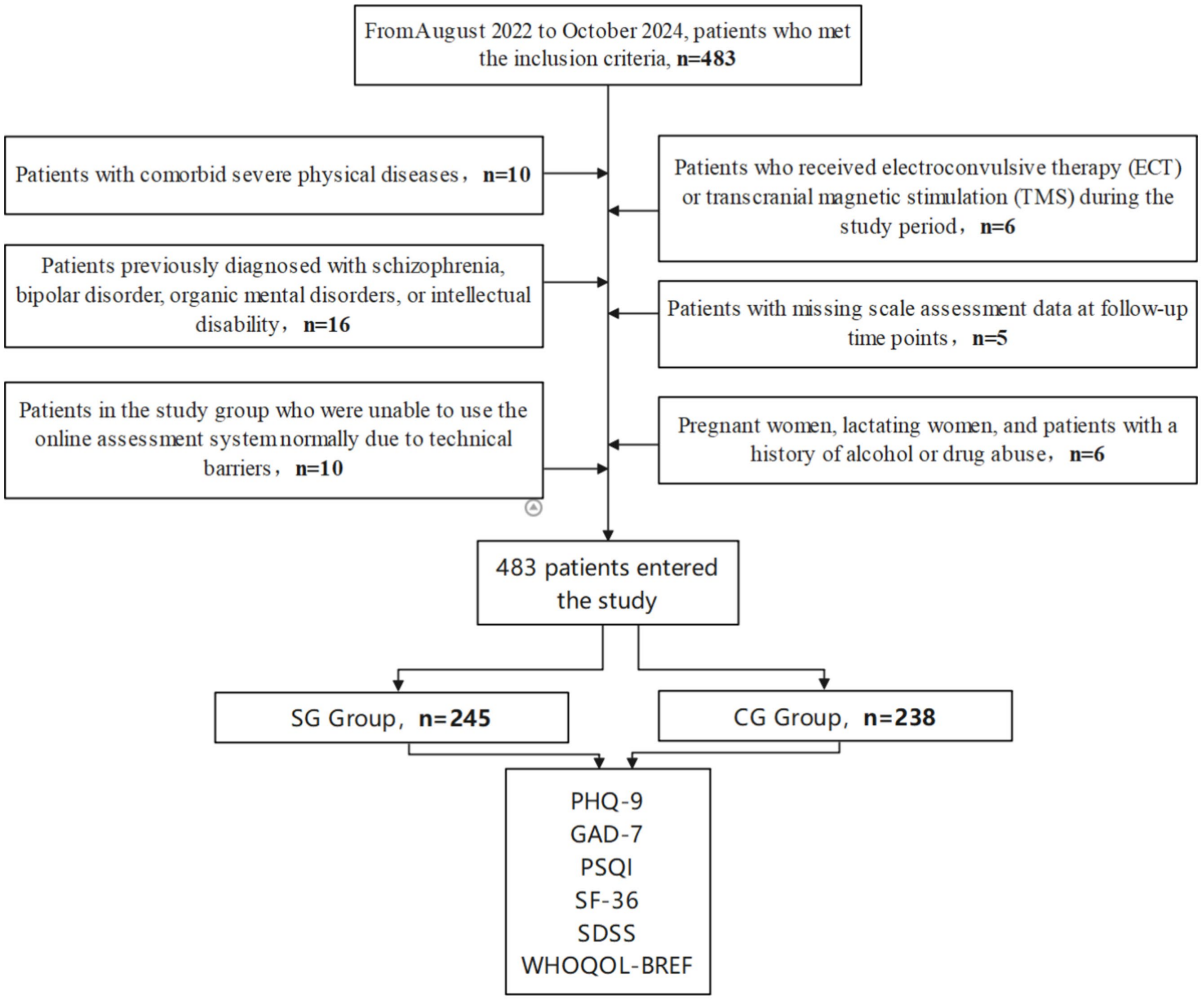
Research design flow chart.

The specific research design flow chart is shown in Figure 1.

### 2.2 Data collection

Using the hospital’s HIT system, the following data were collected: (1) General clinical information, including gender, age, type of psychological disorder, etc.; (2) PHQ-9 scale scores (Ang et al., 2025) (total score 27 points, higher scores indicating more severe depression symptoms), GAD-7 scale (Chamorro-Delmo et al., 2024) (total score 21 points, each item 0–3 points, higher scores indicating more severe anxiety symptoms), and PSQI scale (including dimensions of subjective sleep quality, sleep latency, sleep duration, habitual sleep efficiency, sleep disturbances, use of sleep medication, and daytime dysfunction, total score 21 points, higher scores indicating poorer sleep quality) of both groups at four time points: before intervention, 1 month after intervention, 3 months after intervention, and 6 months after intervention; (3) SF-36 scale (Curelaru and Curelaru, 2024) (8 dimensions: physical functioning, role-physical, bodily pain, general health, vitality, social functioning, role-emotional, and mental health, total score 0–100 points, higher scores representing higher quality of life), SDSS scale (Fischer et al., 2025) (4 dimensions: family role, social interaction, work ability, and self-care, total score 40 points, higher scores indicating more severe social function deficits), and WHOQOL-BREF scale (Xu et al., 2022) (4 dimensions: physical health, psychological health, social relationships, and environment, as well as 2 separate items of overall quality of life and general health, total score 0–100 points, higher scores indicating better quality of life) scores of both groups before intervention and at 6 months after intervention.

To evaluate the clinical meaningfulness, we adopted validated minimal clinical important difference (MCIDs) for all outcomes. We defined PHQ-9 had more than 5-point reduction as the clinical improvement in depression. And meaningful anxiety relief was recognized as more than 4-point reduction of GAD-7. Sleep diary was a daily log capturing sleep onset time, wake-up time number of awakenings, sleep quality rating. Based on the analysis of sleep diary, PSQI score was assessed and more than 3 points reduction indicated a clinical improvement in sleep function. The MCIDs of SF-36 was defined 5-point increase.

### 2.3 Data application

The control group received conventional treatment, including: (1) Pharmacological treatment: Patients with anxiety disorders were treated with selective serotonin reuptake inhibitors (sertraline 50–200 mg/day or paroxetine 20–60 mg/day) or serotonin-norepinephrine reuptake inhibitors (venlafaxine 75–225 mg/day); patients with depressive disorders were treated with antidepressant drugs (fluoxetine 20–60 mg/day or escitalopram 10–20 mg/day); patients with sleep disorders were treated with short-term benzodiazepines or non-benzodiazepine sedative-hypnotics; (2) Psychological treatment: Outpatient psychological counseling was conducted once every 2–4 weeks (45–60 min per session), mainly adopting cognitive behavioral therapy or supportive psychotherapy.

Additionally, the study group received auxiliary management with a multi-component web-based mental health intervention. This system comprises: (1) Online scale assessment module: Patients completed regular self-assessments using scales such as the Patient Health Questionnaire-9 (PHQ-9), Generalized Anxiety Disorder-7 (GAD-7), and Pittsburgh Sleep Quality Index (PSQI) via mobile terminals or personal computers (before intervention, 1 month, 3 months, and 6 months after intervention); (2) Sleep diary function: Patients recorded bedtime, wake-up time, number of awakenings, and sleep quality scores daily; (3) Automatic reminder function: The system regularly sent assessment reminders and treatment adherence prompts; (4) Data visualization feedback: Symptom change trend charts were provided to patients and clinicians; (5) Personalized health education: Targeted mental health knowledge and self-management suggestions were pushed based on the assessment results. The control group completed identical assessments using paper questionnaires administered during clinic visits at the same time intervals.

To reduce the influence of subjective factors in data application, this study assigned three different individuals as data collector, data analyst, and data auditor. The data collector was responsible for collecting data and assigning codes to patients (such as patient 1, patient 2, concealing patient names), the data analyst performed statistical analysis on the collected data, and the data auditor was responsible for data verification. Patient data were protected from leakage to maintain patient privacy. Due to the historical controlled nature and time-separated design of this study, assessor blinding was not feasible.

### 2.4 Statistical analysis

SPSS 24.0 software was used for statistical analysis. For measurement data conforming to normal distribution with homogeneity of variance, *t*-tests were used and described as (mean ± standard deviation). For skewed data or measurement data with unequal variances, non-parametric Mann–Whitney tests (*U* tests) were used and described as median (upper and lower quartiles). Count data were compared using chi-square tests and expressed as *n* (%). As this was a historical controlled study utilizing all available eligible patients during the specified time periods, sample size was not calculated *a priori*. The effect sizes (Cohen’s *d*) for between-group comparisons were calculated, where |*d*| = 0.2 indicates a small effect, |*d*| = 0.5 indicates a moderate effect, and |*d*| = 0.8 indicates a large effect. Considering that this study is an exploratory study, uncorrected *p* values were used for the primary analyses. To control the risk of Type I error caused by multiple comparisons, we performed Bonferroni correction on the primary outcome indicators (between-group comparisons of PHQ-9, GAD-7, and PSQI scores at 6 months post-intervention) (corrected significance level *α* = 0.05/3 ≈ 0.017), and the results still showed statistical significance. *p* < 0.05 was considered statistically significant.

## 3 Results

### 3.1 Comparison of basic demographic data between the two groups

Demographic characteristics including gender distribution, age, and types of psychological disorders were collected from the HIT system for all enrolled patients and compared between groups. Because of the association between obesity and anxiety or depression, we collected the body mass index (BMI) of patients to avoid bias (3). The results indicated no statistically significant differences between the two groups in any demographic characteristics (*p* > 0.05), demonstrating comparability of the two groups at baseline (Table 1).

**Table 1:** Comparison of basic demographic data of enrolled patients (x̄ ± s)/[*n*(%)].

General clinical data		Study group (*n* = 245)	Control group (*n* = 238)	*p*
Gender	Male	112 (45.71)	105 (44.12)	0.724
	Female	133 (54.29)	133 (55.88)	
Mean age (years)		36.11 ± 7.87	36.24 ± 4.58	0.825
Mean BMI (kg/m^2^)		24.33 ± 3.39	24.20 ± 3.86	0.694
Type of psychological disorder	Anxiety disorder	72 (29.39%)	68 (28.57%)	0.998
	Depressive disorder	58 (23.67%)	55 (23.11%)	
	Sleep disorder	42 (17.14%)	39 (16.39%)	
	Adjustment disorder	31 (12.65%)	33 (13.87%)	
	Post-traumatic stress disorder	24 (9.80%)	25 (10.50%)	
	Obsessive-compulsive disorder	18 (7.35%)	18 (7.56%)	
PHQ-9 score		15.42 ± 4.23	15.67 ± 4.15	0.521
GAD-7 score		13.28 ± 3.87	13.51 ± 3.94	0.503
PSQI score		12.76 ± 3.56	12.93 ± 3.62	0.603

### 3.2 Comparison of depression scale (PHQ-9) scores between the two groups

**Figure 2:**
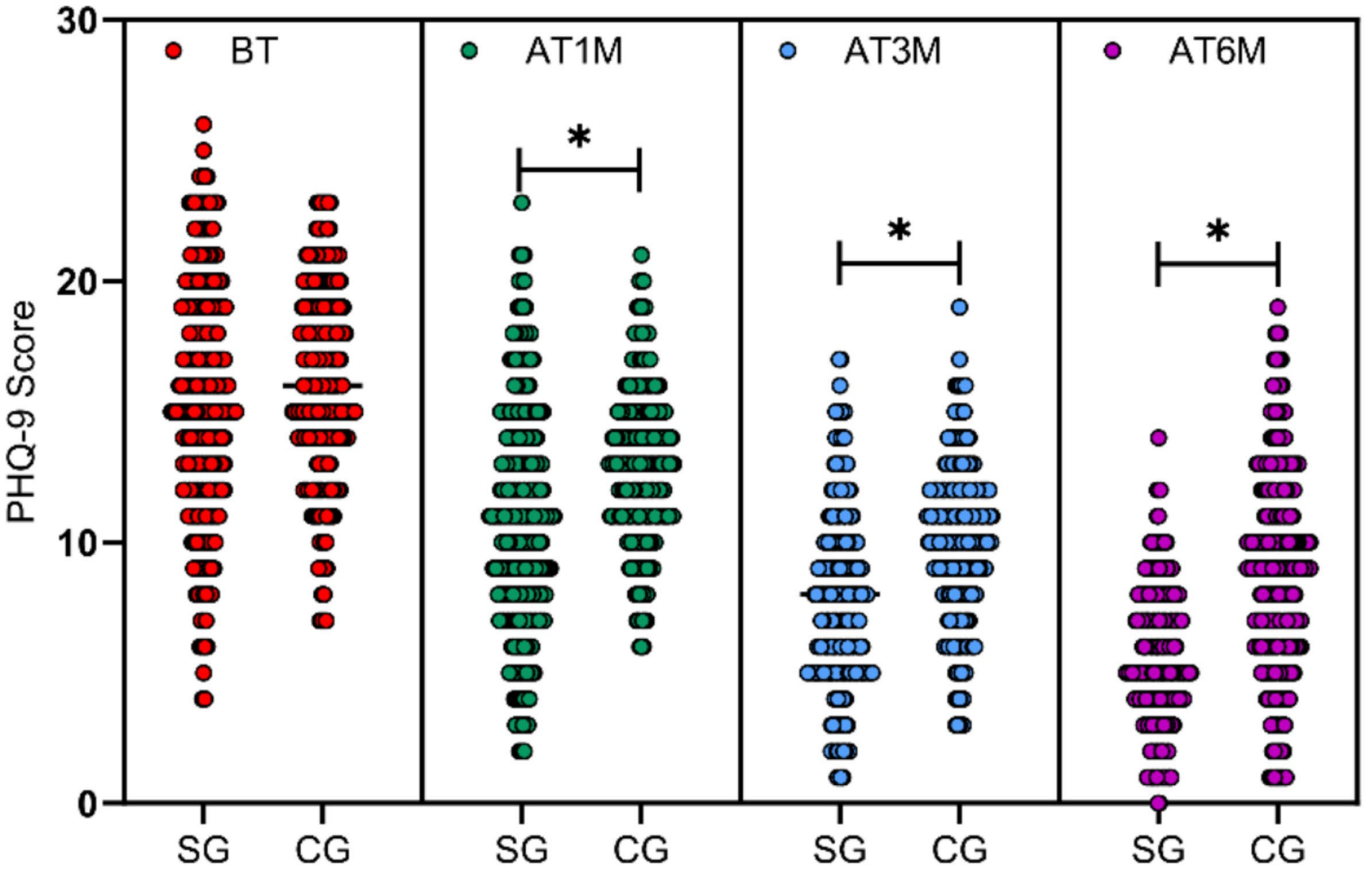
Differences in depression scale scores between the two groups before and after intervention. PHQ-9 scores at baseline (BT) and at 1 month (AT1M), 3 months (AT3M), and 6 months (AT6M) post-intervention. No significant difference was observed between the study group (SG) and control group (CG) at baseline (p > 0.05). At all post-intervention time points, the study group exhibited significantly lower PHQ-9 scores than the control group (*p* < 0.05, marked with *).

PHQ-9 scale scores were assessed at baseline and at 1, 3, and 6 months post-intervention for both groups. Baseline PHQ-9 scores did not differ significantly between groups (*p* > 0.05). At 1 month, 3 months, and 6 months post-intervention, the study group exhibited significantly lower PHQ-9 scores compared to the control group (*t* = 5.987, *p* < 0.001, Cohen’s *d* = 0.55; *t* = 11.526, *p* < 0.001, Cohen’s *d* = 1.05; *t* = 14.852, *p* < 0.001, Cohen’s *d* = 1.38), with statistically significant between-group differences at all three follow-up time points (*p* < 0.05), as shown in Figure 2.

### 3.3 Comparison of anxiety scale (GAD-7) scores between the two groups

**Figure 3:**
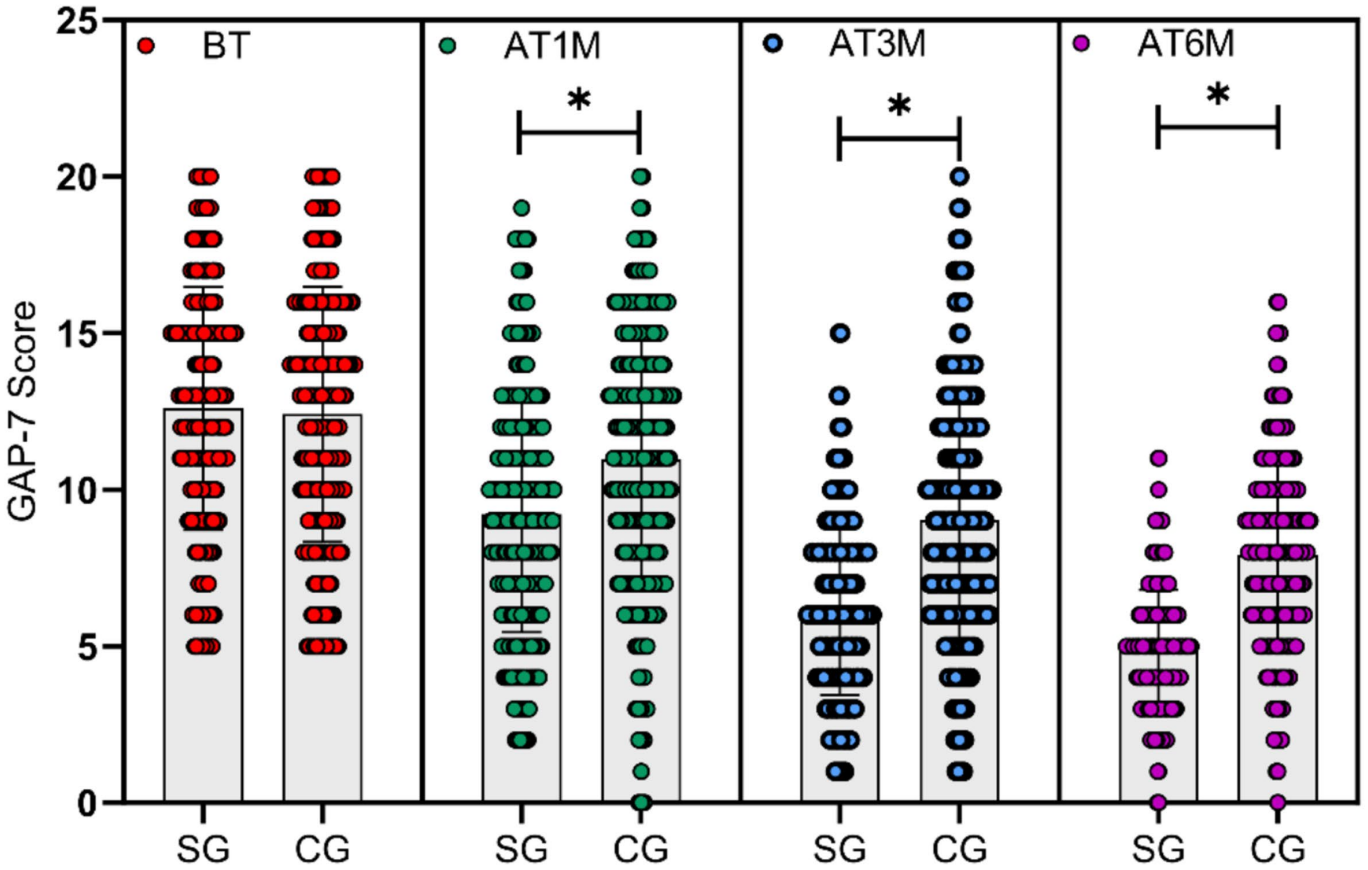
Comparison of GAD-7 scores between the two groups at different time points. GAD-7 scores at baseline (BT) and at 1 month (AT1M), 3 months (AT3M), and 6 months (AT6M) post-intervention. No significant difference was observed between the study group (SG) and control group (CG) at baseline (p > 0.05). At all post-intervention time points, the study group exhibited significantly lower GAD-7 scores than the control group (*p* < 0.05, marked with *).

GAD-7 scale scores were assessed at baseline and at 1, 3, and 6 months post-intervention for both groups. Baseline GAD-7 scores were comparable between groups (*p* > 0.05). At 1 month, 3 months, and 6 months post-intervention, the study group exhibited significantly lower GAD-7 scores compared to the control group (*t* = 5.876, *p* < 0.001, Cohen’s *d* = 0.54; *t* = 10.547, *p* < 0.001, Cohen’s *d* = 0.97; *t* = 14.652, *p* < 0.001, Cohen’s *d* = 1.36), with statistically significant between-group differences at all three follow-up time points (*p* < 0.05), as shown in Figure 3.

### 3.4 Comparison of Pittsburgh sleep quality index (PSQI) scores between the two groups

**Figure 4:**
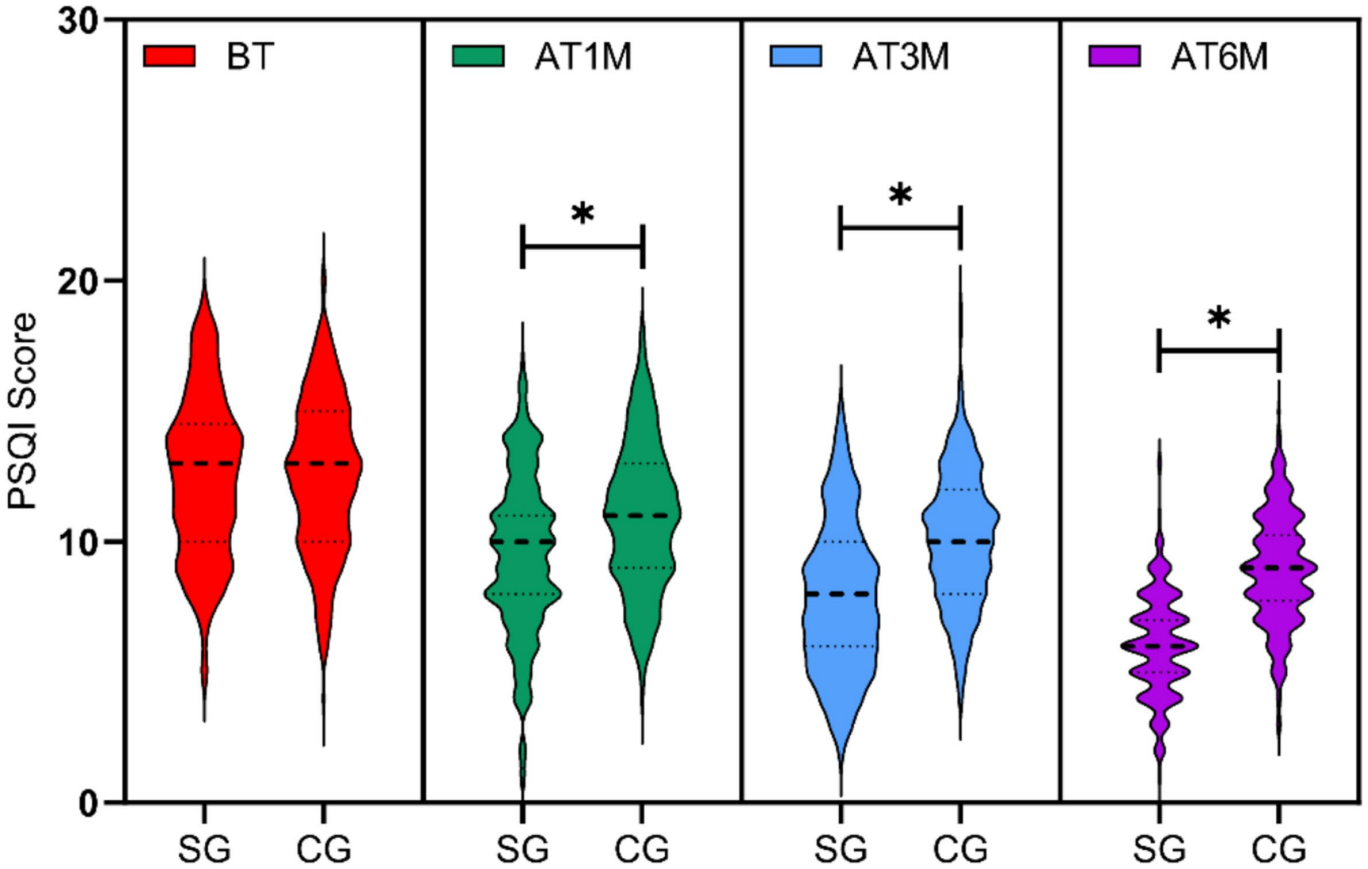
Comparison of PSQI scores between the two groups at different time points. Sleep quality assessed by PSQI at baseline (BT) and 1 month (AT1M), 3 months (AT3M), and 6 months (AT6M) post-intervention. SG, study group; CG, control group. *p < 0.05 vs. control group at the same time point. Lower scores indicate better sleep quality.

PSQI scale scores were assessed at baseline and at 1, 3, and 6 months post-intervention for both groups. Baseline PSQI scores showed no significant difference between groups (*p* > 0.05). At 1 month, 3 months, and 6 months post-intervention, the study group demonstrated significantly lower total PSQI scores compared to the control group (*t* = 5.234, *p* < 0.001, Cohen’s *d* = 0.48; *t* = 9.012, *p* < 0.001, Cohen’s *d* = 0.83; *t* = 13.456, *p* < 0.001, Cohen’s *d* = 1.25), with statistically significant between-group differences at all three follow-up time points (*p* < 0.05), as shown in Figure 4.

### 3.5 Comparison of quality of life (SF-36) scores between the two groups

**Figure 5:**
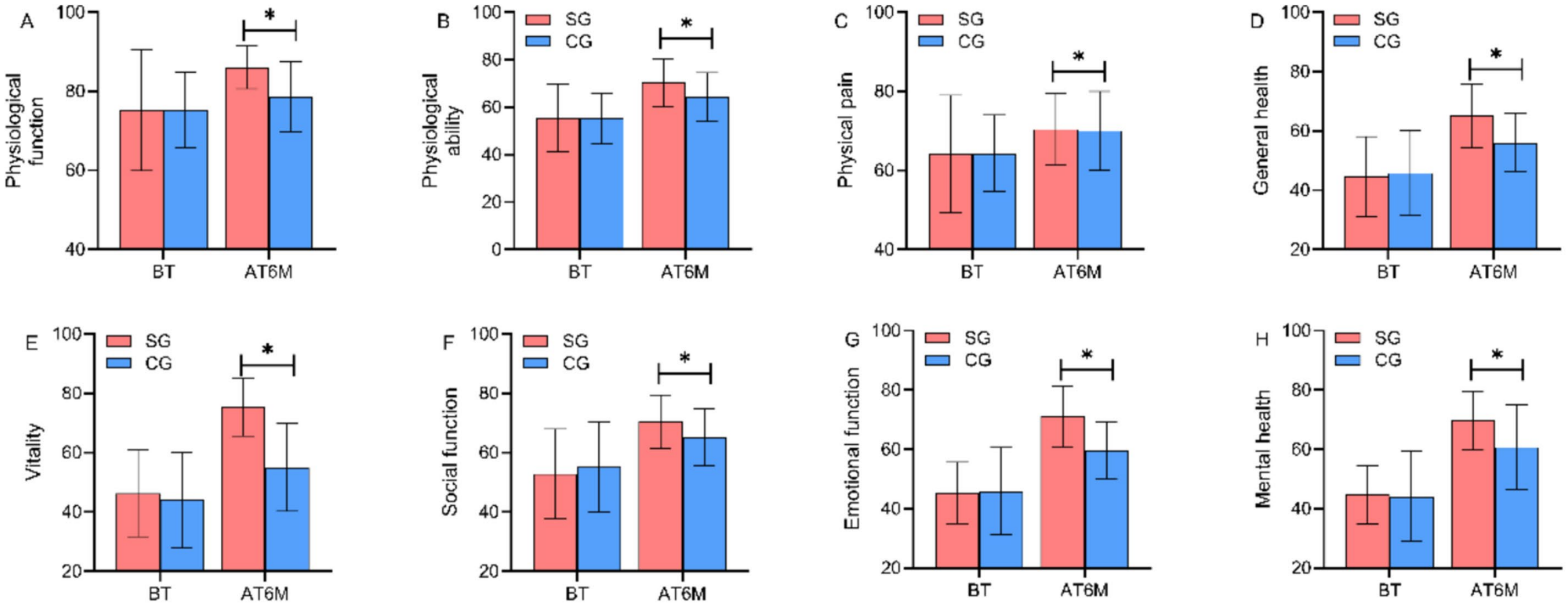
Comparison of SF-36 dimension scores between the two groups before and after intervention. Health-related quality of life assessed by SF-36 at baseline (BT, left panel) and 6 months post-intervention (AT6M, right panel). Eight dimensions: physical functioning (PF), role-physical (RP), bodily pain (BP), general health (GH), vitality (VT), social functioning (SF), role-emotional (RE), and mental health (MH). SG, study group; CG, control group. *p < 0.05 vs. control group at the same time point. Higher scores indicate better quality of life.

SF-36 scale scores were assessed at baseline and at 6 months post-intervention for both groups to evaluate health-related quality of life across eight dimensions: physical functioning, role-physical, bodily pain, general health, vitality, social functioning, role-emotional, and mental health. Baseline SF-36 scores across all dimensions showed no significant differences between groups (*p* > 0.05). At 6 months post-intervention, the study group demonstrated significantly higher scores in all eight SF-36 dimensions compared to the control group, with statistically significant between-group differences (*p* < 0.05). The total SF-36 score in the study group at 6 months post-intervention was 74.53 ± 9.94, significantly higher than the control group’s score of 63.70 ± 9.89 (*t* = 11.997, *p* < 0.001, Cohen’s *d* = 1.09), as shown in Figure 5.

### 3.6 Comparison of social functioning (SDSS) and overall quality of life (WHOQOL-BREF) scores between the two groups

**Figure 6:**
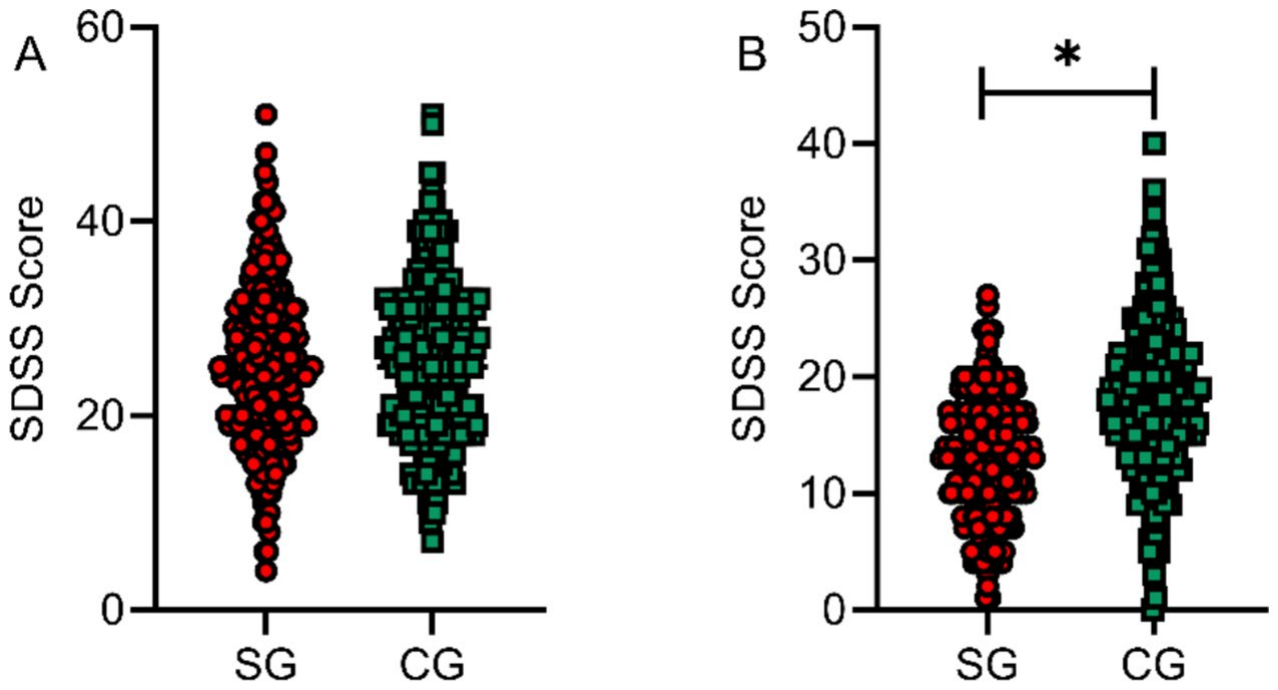
Comparison of SDSS scores between the two groups before and after intervention. Social functioning assessed by SDSS at baseline **(A)** and 6 months post-intervention **(B)**. SG, study group; CG, control group. *p < 0.05 vs. control group. Lower scores indicate better social functioning (less social disability).

SDSS scale scores were assessed at baseline and at 6 months post-intervention for both groups to evaluate social functioning across four dimensions: family role, social interaction, work ability, and self-care. Baseline SDSS scores showed no significant difference between groups (*p* > 0.05). At 6 months post-intervention, the study group demonstrated significantly lower SDSS scores compared to the control group, with statistically significant between-group differences (*t* = 11.324, *p* < 0.001, Cohen’s *d* = 1.06), indicating improved social functioning, as shown in Figure 6.

**Figure 7:**
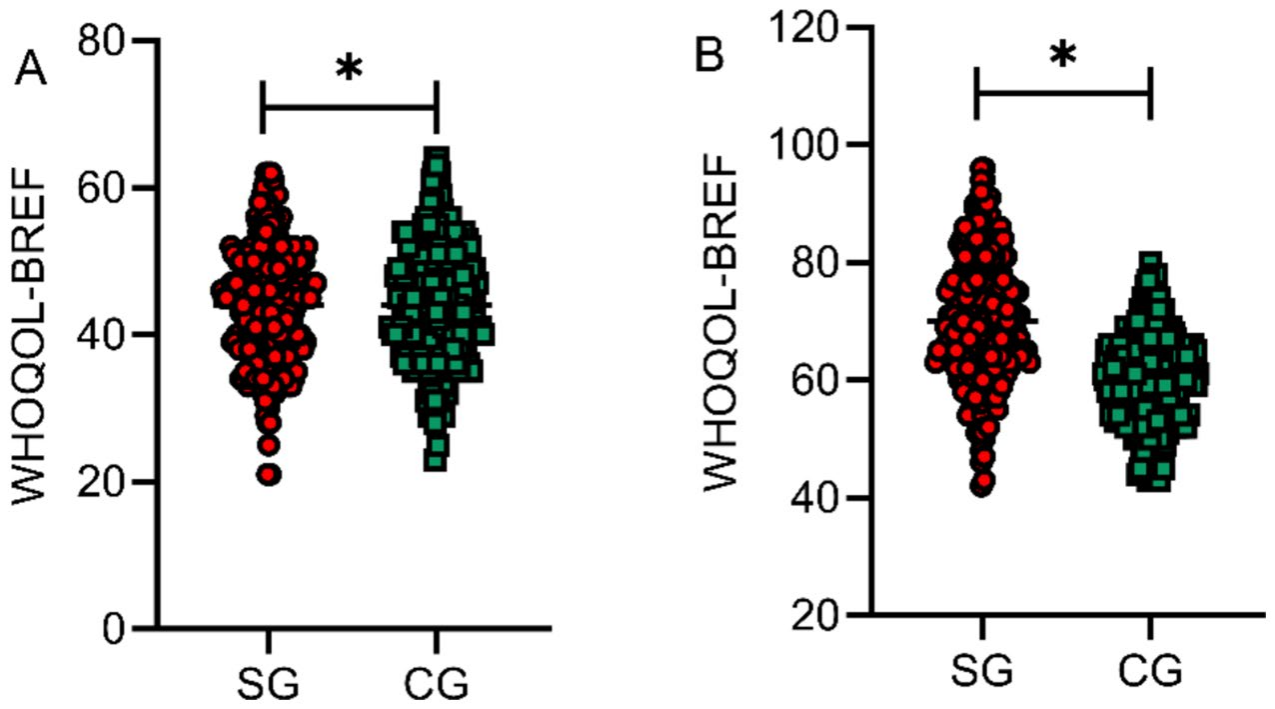
Comparison of WHOQOL-BREF scores between the two groups before and after intervention. Overall quality of life assessed by WHOQOL-BREF at baseline **(A)** and 6 months post-intervention **(B)**. SG, study group; CG, control group. *p < 0.05 vs. control group. Higher scores indicate better quality of life.

WHOQOL-BREF scale scores were also assessed at baseline and at 6 months post-intervention for both groups to evaluate overall quality of life across four domains: physical health, psychological health, social relationships, and environment. Baseline WHOQOL-BREF scores showed no significant difference between groups (*p* > 0.05). At 6 months post-intervention, the study group demonstrated significantly higher WHOQOL-BREF scores compared to the control group, with statistically significant between-group differences (*t* = 11.236, *p* < 0.001, Cohen’s *d* = 1.03), indicating enhanced overall quality of life, as shown in Figure 7.

### 3.7 Comparison of clinical remission rate between the two groups

Based on the MCID criteria, we compared the proportions of patients with clinically significant improvements in the two groups at 6 months post-intervention. The clinical response rates in the study group were significantly higher than those in the control group for depressive symptoms, anxiety symptoms, and sleep quality (all *p* < 0.05). Similarly, the study group also had higher achievement rates of quality-of-life improvement and social function improvement compared with the control group, as shown in Table 2.

**Table 2:** Comparison of clinical remission rate (x̄ ± s)/[*n*(%)].

Score	MCID	Study group (*n* = 245)	Control group (*n* = 238)	*χ* ^2^	*p*
PHQ-9	Decreased ≥ 5	178 (72.65%)	142 (59.66%)	9.35	0.002
GAD-7	Decreased ≥ 4	185 (75.51%)	148 (62.18%)	11.02	0.001
PSQI	Decreased ≥ 3	192 (78.37%)	156 (65.55%)	10.58	0.001
SF-36	Increased ≥ 5	195 (79.59%)	151 (63.45%)	16.42	<0.001

## 4 Discussion

This study systematically evaluated the association between the multi-component web-based mental health intervention and clinical outcomes through a historical controlled design involving 483 patients across six common psychological disorders. Results demonstrated that patients receiving treatment assisted by the web-based system (July 2023–October 2024) achieved significantly greater improvements in depression symptoms (PHQ-9), anxiety symptoms (GAD-7), and sleep quality (PSQI) compared to those receiving conventional treatment (August 2022–June 2023), with benefits evident from 1 month post-intervention and sustained through 6-month follow-up. Moreover, the intervention group exhibited superior quality of life (SF-36 and WHOQOL-BREF) and social functioning (SDSS), suggesting that digital mental health assessment may provide multidimensional clinical benefits beyond core symptom reduction.

The observed associations across multiple outcome domains may plausibly relate to several features of the multi-component web-based system. First, real-time data collection and visualization enable clinicians to dynamically monitor symptom trajectories and adjust interventions promptly, facilitating precision medicine approaches that traditional episodic assessments cannot achieve. Second, automated reminder functions and accessible self-assessment platforms enhance treatment adherence by reducing logistical barriers and maintaining continuous patient engagement between clinic visits. Third, the anonymity and privacy features of digital platforms may reduce stigma-related concerns, encouraging more honest symptom reporting and deeper therapeutic engagement, as noted by research on network-based interventions (Kim et al., 2023), which found that such approaches help identify and support psychologically vulnerable groups online. Fourth, the convenience of anytime-anywhere assessment improves accessibility for patients with mobility limitations, geographic constraints, or scheduling conflicts, addressing a critical gap in traditional mental health service delivery. However, given the historical nature of the comparison, these potential mechanisms should be regarded as speculative and intended to inform future hypothesis-driven research rather than to explain definitive intervention effects.

The significant reduction in depression symptoms observed in this study is consistent with previous research on digital assessment platforms for mental health (Martin-Key et al., 2021), which demonstrated that such systems enhance clinical decision-making and patient engagement. The personalized feedback and cognitive restructuring elements embedded in our web-based system may have contributed to observed differences by potentially supporting patients in identifying negative thought patterns and develop adaptive coping strategies. Similarly, the substantial decrease in anxiety symptoms aligns with a prospective cohort analysis (Li et al., 2021) showing that digital mental health assessment enables early identification and active intervention for individuals with anxiety problems. The anonymity feature of our system may have been particularly beneficial for patients with social anxiety, reducing the interpersonal pressure inherent in face-to-face assessments, as suggested by research on mental health assessment tools (Newson and Thiagarajan, 2020).

Sleep quality improvements in our study parallel findings from research conducted during the COVID-19 pandemic (Fazia et al., 2023), which demonstrated that online mindfulness interventions significantly improved sleep quality and emotional regulation. The sleep diary function in our web-based system provided clinicians with longitudinal sleep pattern data, which may have informed clinical decision-making such as sleep hygiene education and relaxation training, consistent with approaches validated by studies on online psychoeducation and self-compassion interventions (Chinvararak et al., 2024; Sheng et al., 2023). Research on online behavioral self-help interventions (Elder et al., 2024) found that such approaches rapidly improved acute insomnia severity, supporting our finding of early improvement at 1-month follow-up. However, we observed less pronounced improvement in the “use of sleep medication” dimension, suggesting that modifying medication-related behaviors may require longer-term intervention, as noted by a comparative study of online mindfulness and relaxation interventions (Pickett et al., 2024).

The enhancement in quality of life and social functioning represents a particularly important finding, as it suggests that observed differences extend beyond symptom measures to meaningful improvements in daily functioning. This aligns with a systematic review (White et al., 2022) which found that online psychological interventions effectively reduce symptoms of depression, anxiety, and general distress in patients with chronic health conditions while improving overall well-being. Our intervention group showed the most substantial improvements in the “social functioning” and “role-emotional” dimensions of SF-36, likely reflecting enhanced capacity to manage interpersonal relationships and emotional challenges through continuous digital support. The significant reduction in SDSS scores, particularly in “social interaction” and “work ability” dimensions, is consistent with a meta-analysis (Shi et al., 2024) demonstrating that internet-based interventions improve both clinical symptoms and social functioning in patients with pathological health anxiety.

Compared to previous research, this study offers several unique contributions. Most prior digital mental health studies focused on single diagnostic categories, whereas we enrolled patients with six common psychological disorders, providing preliminary evidence for the broad applicability of web-based assessment across diverse psychiatric conditions. Additionally, while many studies evaluated single outcome measures, we employed a comprehensive battery assessing core symptoms, quality of life, and social functioning, offering a more complete picture of clinical impact. The relatively rapid onset of benefits observed in our study—with significant improvements evident at 1 month—may reflect the integrated design of our system, which combines standardized assessment with automated feedback and personalized intervention recommendations, rather than functioning solely as a measurement tool.

From a clinical practice perspective, these findings have important implications for mental health service delivery. The multi-component web-based mental health intervention may be feasibly integrated into existing clinical workflows, enabling routine screening and monitoring without substantial increases in clinician workload. For healthcare systems facing resource constraints, such digital tools may support more efficient resource allocation by identifying patients requiring intensive intervention while supporting self-management in those with milder symptoms. The accessibility features are particularly valuable for underserved populations, including those in remote areas or with limited mobility. However, successful implementation requires attention to digital literacy training, technical support infrastructure, and strategies to address potential digital divide issues that could exclude vulnerable populations.

It is important to interpret the present findings in light of several methodological considerations. Most notably, this study employed a historical controlled design, with the control group recruited during the COVID-19 period and the intervention group enrolled after the easing of pandemic-related restrictions. This temporal separation coincided with substantial changes in social conditions, healthcare accessibility, treatment-seeking behavior, and population-level psychological stressors. As a result, differences observed between groups may reflect not only the introduction of the web-based intervention, but also broader secular trends and contextual changes associated with the transition from the pandemic to the post-pandemic period. Although baseline demographic characteristics and symptom scores were comparable between groups, such similarities cannot fully account for unmeasured time-dependent confounding inherent in historical designs. Factors such as delayed care during the pandemic, evolving clinical practices, changes in staff experience, and shifts in patient expectations may have influenced outcomes independently of the intervention. Consequently, the study design does not permit clear separation of intervention-related effects from contemporaneous external influences, and causal inference cannot be established. Additional limitations should also be acknowledged. The single-center setting may limit generalizability, follow-up was restricted to 6 months, and concurrent treatments were not standardized across time periods. Although Bonferroni correction was applied to primary outcomes, secondary analyses were not adjusted for multiple comparisons, raising the possibility of Type I error. Taken together, these factors underscore the need for cautious interpretation of the findings.

Future research should prioritize prospective randomized controlled trials with concurrent group allocation to eliminate historical bias and enable stronger causal inference. Such studies should include comprehensive baseline assessments across all outcome measures, standardized concurrent treatments, assessor blinding, and extended follow-up periods (12–24 months minimum) to evaluate sustained effectiveness. Multi-center trials would enhance generalizability, while subgroup analyses could identify which patient populations benefit most from digital assessment. Economic evaluations comparing cost-effectiveness to traditional care models would inform implementation decisions. Additionally, research should explore optimal integration strategies with evidence-based psychotherapies, customization for special populations (elderly, adolescents, those with limited digital literacy), and examination of mediators and moderators of treatment response to understand mechanisms of action and personalize intervention delivery.

In conclusion, this historical controlled study provides preliminary, observational evidence of an association between a multi-component web-based mental health intervention and improvements in symptom severity, quality of life, and social functioning among patients with psychological disorders. Given the substantial risk of historical confounding related to the COVID-19 and post-pandemic transition, these findings should be interpreted as exploratory and hypothesis-generating, rather than as evidence of causal effectiveness. Prospective randomized controlled trials with concurrent group allocation are required to determine whether the observed associations reflect true intervention effects.

## Data Availability

The clinical data used to support the findings of this study are available from the corresponding author upon reasonable request, subject to ethical approval and patient privacy protection.
